# Alcohol devaluation has dissociable effects on distinct components of alcohol behaviour

**DOI:** 10.1007/s00213-018-4839-2

**Published:** 2018-02-26

**Authors:** Abigail K Rose, Kyle Brown, James MacKillop, Matt Field, Lee Hogarth

**Affiliations:** 1 Department of Psychological Sciences, University of Liverpool, Eleanor Rathbone Building, Bedford Street South, Liverpool, L69 7ZA UK; 2 UK Centre for Tobacco and Alcohol Studies, Liverpool, UK; 3 Faculty of Business, Law, and Social Sciences, Birmingham City University, Birmingham, UK; 4 Peter Boris Centre for Addictions Research, McMaster University, Hamilton, ON Canada; 5 Department of Psychology, University of Exeter, Exeter, UK

**Keywords:** Alcohol choice, Alcohol consumption, Attentional bias, Devaluation, Value-based decision-making, Goal-directed behaviour, Cue-elicited behaviour

## Abstract

**Rationale:**

Substance-related behaviour is often viewed as an appetitive behaviour, motivated by the reinforcing effects of the drug. However, there are various indices of substance motivation (e.g. attentional bias, behavioural economic demand, craving) and it is unclear how these are related or whether they play an important role in all types of substance-related behaviour.

**Objectives:**

(1) To determine the effect of alcohol devaluation on several indices of alcohol motivation and goal-directed and cue-elicited alcohol behaviour. (2) To investigate which components of motivation mediate any effect of devaluation on behaviour.

**Methods:**

Sixty-two social drinkers gave baseline measures of alcohol craving, behavioural economic demand and choice for alcohol vs. soft drink. Participants tasted alcohol which was either unadulterated (control) or adulterated with a bitter solution (devaluation) before craving and demand were measured again. Alcohol choice was assessed in several phases: extinction (evaluating goal-directed behaviour), in the presence of drink cues (Pavlovian-to-instrumental transfer (PIT, cue-elicited behaviour)), and reacquisition. Attentional bias (AB) was measured by tracking eye movements towards the drink cues during novel PIT trials where both cues were presented. Finally, consumption was evaluated in a taste test.

**Results:**

Alcohol devaluation reduced alcohol-related demand, AB, alcohol choice in all phases, and consumption. Alcohol cues presented during PIT increased alcohol choice above baseline irrespective of devaluation. AB and demand for alcohol fully mediated the effect of devaluation on alcohol choice during extinction, AB fully mediated the effect on cue-elicited (specific PIT) alcohol choice and alcohol consumption.

**Conclusions:**

Alcohol behaviour in social drinkers is largely sensitive to devaluation, i.e. governed by current motivational value of the drug (suggesting goal-directed behaviour). However, a dissociable form of stimulus control can also drive alcohol-seeking independently of drug value (specific PIT). Mediation analyses suggests that AB may play a paradoxical role in both forms of alcohol seeking and consumption.

**Electronic supplementary material:**

The online version of this article (10.1007/s00213-018-4839-2) contains supplementary material, which is available to authorized users.

## Introduction

Clarifying the processes that determine drug choice and consumption remains an active research enterprise and one which may inform development of more effective treatments. Evidence has identified a number of indices of motivation which are likely to be involved in drug-related behaviour, including attentional bias (AB), craving, economic demand and concurrent choice. However, the importance of these indices in terms of driving drug behaviours is less clear.

Within an associative learning framework, behaviour may occur through two systems: goal-directed (or model-based) and habitual (or model-free). Goal-directed processes involve a representation of the drug and its current value. If the drug’s value is high, relative to alternative rewards, this will trigger intentional drug seeking (Hogarth et al. 2013a; Sebold et al. 2016). It is reasonable, therefore, to assume that motivational processes (e.g. AB, craving, economic demand) will be involved in goal-directed behaviours. Indeed, a number of studies have found positive relationships between these indices and drug use and clinical symptoms and that these measures can predict use and relapse (e.g. Mackillop et al. 2010; Marhe et al. 2013; Tiffany and Wray 2012; Townshend and Duka 2001).

Although their causal role is unclear, these motivational indices may nonetheless contribute to drug behaviours. A recent review suggests that they reflect an underlying appetitive motivational process, and that it is the current *value* of the drug which primarily drives drug choice and consumption (see Field et al. 2016). According to this framework, value has not only direct influence on drug use but also indirect effects via reciprocal relationships with AB, craving and motivational conflict (which would involve economic demand). Our previous work has found that increasing or decreasing the value of the drug can modify AB (reviewed in Field et al. 2016), craving (Rose et al. 2013), goal-directed concurrent choice (e.g. reviewed in Hogarth et al. 2013a) and economic demand (Acker and Mackillop 2013). The current study is the first to bring these measures together to identify their role in different aspects of alcohol-related behaviour using a devaluation paradigm.

Although a substantial evidence base exists demonstrating sensitivity to devaluation procedures, outcome-related stimuli seem able to override this effect. The key example of this comes from Pavlovian-to-instrumental transfer (PIT) paradigms. PIT describes the process by which a reward-associated cue stimulates reward-seeking behaviour, even though the cue and response may have never previously co-occurred. Importantly, the ability of a drug cue to prime a separately trained drug-seeking response appears unaffected by drug devaluation (e.g. Hitsman et al. 2013). To explain this dissociation, dual-process accounts of drug motivation have been expressed from the perspective of neuroscience (Robinson and Berridge 2008), behavioural automaticity (Tiffany 1990), cognitive psychology (Wiers et al. 2007) and learning theory (Hogarth et al. 2013a). These accounts suggest that dissociable forms of drug-seeking control can operate simultaneously to determine drug-seeking (Hogarth et al. 2013a).

We previously used a novel devaluation paradigm to assess concurrent choice, and AB for alcohol and soft drink rewards, before and after devaluing alcohol in non-dependent drinkers (Rose et al. 2013). We found reductions in alcohol choice and AB for alcohol cues following devaluation, suggestive of goal-directed behaviour. Furthermore, mediation analysis suggested that AB accounted for ~ 30% of the reduction in alcohol choice following devaluation. The current study therefore provides the opportunity to replicate this finding, as well as extend it by identifying the mediating role of multiple motivational components (AB, craving, economic demand) in any devaluation effect on goal-directed alcohol behaviour (choice and consumption). In addition, the current paradigm included a specific PIT test in which choice between an alcohol and soft drink response was made in the presence of either the alcohol or soft drink cue (Martinovic et al. 2014). If goal-directed and cue-elicited responding can co-occur, cue-priming of the alcohol response should be unaffected by alcohol devaluation.

In summary, the current study sought to clarify and extend previous findings concerning processes underlying alcohol-related behaviour. Firstly, we tested whether alcohol devaluation would impact several outcome measures: AB, behavioural economic demand, craving, goal-directed alcohol choice, cue-elicited alcohol choice (specific PIT test) and consumption. We hypothesised that all measures would show a devaluation effect with the exception of cue-elicited choice. Secondly, the study determined whether our measures of motivation mediated any devaluation effect on alcohol-related behaviour (choice, consumption). The analysis helps clarify associative learning theories of addictive behaviour and the conditions under which the two controllers operate, and highlights key components of motivation which drive alcohol behaviour.

## Materials and methods

### Participants

Sixty-two social drinkers (29 females) aged 22.35 years (SD ± 4.24) were recruited. Inclusion criteria were self-reported good general/psychiatric health, no history of alcohol or drug abuse/dependence, no current use of medication affected by alcohol, no desire to reduce consumption, and females could not be pregnant. Participants were invited to take part only if they consumed alcohol on a weekly basis (weekly unit [8 g of alcohol] consumption: M = 27.12 [± 16.48]), and reported lager and cola or lemonade as a preferred alcohol/soft drink, respectively (the drinks used in the study). This study received approval from the University of Liverpool Research Ethics Committee.

### Self-report measures

*Alcohol Use Disorders Identification Test* (AUDIT) (Saunders et al. 1993) identifies hazardous and harmful alcohol use across 10 items.

*Time Line Follow Back* (TLFB) (Sobell and Sobell 1992) assesses typical weekly alcohol consumption and binge frequency (female = ≥ 6 units p/drinking episode, males = ≥ 8 units p/drinking episode) using a diary format.

*Alcohol Urge Questionnaire* (AUQ) (Bohn et al. 1995) produces a single craving score assessing desire for alcohol, expectation of positive effect from drinking, and inability to avoid drinking if alcohol was available.

*Alcohol Purchase Task* (Murphy and MacKillop 2006) a behavioural economic measure of alcohol demand. Participants are asked to imagine a typical drinking scenario in which their preferred (unadulterated) alcoholic drink is available (a standard pint of beer or glass of wine). They are asked to estimate their alcohol consumption across 25 escalating prices, ranging from £0 to £15 per drink. Alcoholic drinks could be the participant’s favourite drink from a typical pint of beer, glass of wine, or shot of spirit (with/without mixer). An observed values approach was used to generate most indices of demand: (a) intensity of demand, level of consumption at minimal price; (b) *O*_max_, maximum expenditure for alcohol across prices; (c) *P*_max_, the price at which demand shifts from being inelastic to elastic, which corresponds with the price at which *O*_max_ is reached; (d) breakpoint, the first price that suppresses consumption to zero and (e) elasticity of demand, sensitivity to price as it increases. Hursh and Silberberg’s (2008) exponential demand equation, log_10_*Q* *=* log_10_*Q*_0_ + *k*(*e*^−e*Q0C*^ − 1), was used to create an index of elasticity, where *Q =* consumption at the given price; *Q*_0 *=*_ consumption at 0; *k =* range of the dependent variable (drinks) in logarithmic units, in this case 4; *C =* cost (price) and e = elasticity (rate of decline in log consumption based on price increase).

### Alcohol-seeking behaviour

#### Concurrent choice behaviour

**Fig. 1:**
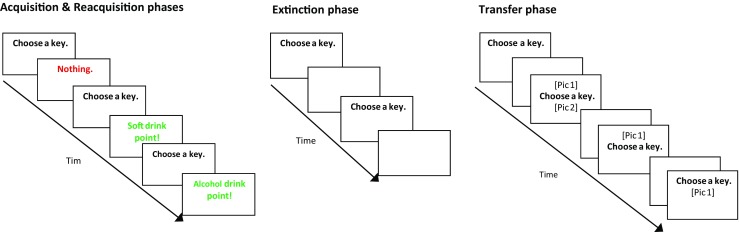
Details of the concurrent choice procedure. All phases required subjects to choose one of two responses to ‘win’ points towards alcohol or soft drinks. Phases differed according to (a) whether feedback regarding their choice was presented onscreen after pressing a key (acquisition and reacquisition phases) and (b) whether alcohol, soft drink or both cues were presented (transfer phase). Attentional bias was measured during trials when both picture cues were present

Hogarth and Chase (2011) (Fig. 1) assessed the relative value of preferred alcohol (lager) to soft drink (cola/lemonade) across several phases (acquisition, extinction, PIT, reacquisition) and has been described in detail elsewhere (see Hogarth et al. 2013b). In each trial, participants were required to select one of two responses (D/H key) on a keyboard to win points for the alcohol or soft drink, respectively. Participants were told that more points would grant them more of that reward which they could take home at the end of the experiment (i.e., unadulterated alcohol or soft drink). However, this was a deception: at the end of the study, all participants were compensated £10 for their time.

#### Acquisition (40 trials)

In each trial, an instruction appeared ‘choose a key’, i.e. D or H. Responding yielded the outcome ‘You win an alcoholic drink point’ or ‘You win a soft drink point’. The response-outcome contingencies were counterbalanced between subjects. The responses were non-deterministic, i.e. on 50% of trials, each response did not produce its respective outcome, but instead produced the outcome ‘You win nothing’. Outcome texts were presented for 2000 ms prior to a random inter-trial interval (750–1000 ms).

#### Nominal extinction (40 trials)

Following drinking manipulation (see below), participants chose between the alcohol and soft drink responses as before but no outcomes were displayed. Choice was expected to be sensitive to devaluation as demonstrated previously, suggesting choice is goal-directed.

#### Outcome-specific Pavlovian-to-instrumental transfer (120 trials)

This phase was identical to the extinction phase, except that prior to each choice, an alcohol cue, soft drink cue, both cues or no cue (30 trials each, randomised presentation, in both cue trials cue location was counterbalanced) was presented. Cues (184 × 171 px) depicted the participant’s preferred alcohol (lager) and non-alcoholic drink (lemonade/cola). Cues were presented above and/or below the centre of the screen for 2000 ms simultaneously with the prompt ‘choose a key’. No outcomes were presented. In the unique PIT trials, where both alcohol and soft drink cues were presented together, eye movements were measured to index attentional bias.

#### Reacquisition (40 trials)

The acquisition phase was repeated to examine whether reintroducing the outcomes would increase the magnitude of the devaluation effect relative to the extinction and PIT phases.

### Devaluation/no-devaluation treatment

Following acquisition (before nominal extinction), participants consumed 30 ml of Becks lager which was unadulterated in the sip-control condition or adulterated with 0.6 ml of bitrex (0.256% solution) in the devaluation condition. Bitrex is used commercially to create a bitter-tasting liquid and this volume has previously established taste aversion in humans (Dwyer et al. 2004). The dose of alcohol (30 ml) is below the threshold required to produce a priming effect greater than placebo (Rose and Duka 2006). Therefore, the 30 ml of unadulterated alcohol was used as a control condition (Rose et al. 2013).

### Attention

Attention to the drink cues during ‘both cue’ PIT trials was measured with an ASL-6000 remote eye-tracker (sampling rate 120 Hz). This measure has been described in detail elsewhere (Rose et al. 2013) and produces two factors: initial fixation (alcohol/soft drink) and dwell time by calculating the proportion of observations to the alcohol, relative to the soft drink, picture (Field et al. 2004). For mediation analysis, a composite attentional bias variable was computed by averaging the two percentage scores as they were comparable (Rose et al. 2013).

### Alcohol consumption

*Bogus taste test* (Jones et al. 2011) Following the choice task, we measured alcoholic vs. soft drink consumption. Participants were given 275 ml of Becks lager and lemonade/cola (i.e., drinks available during the choice task). Unknown to the participant, Becks alcohol-free lager was used to avoid alcohol priming effects caused by the acute effects of alcohol (Rose and Duka 2006). Previous work has shown that participants cannot taste any difference between Becks alcohol and alcohol-free lager (Jones et al. 2011). Participants were asked to rate both drinks on visual analogue scales covering four taste variables (pleasant-unpleasant, flat-gassy, bitter-sweet and tasteless-strong tasting) and were informed that they could consume as much as they liked to make accurate ratings.

### Procedure

**Fig. 2:**
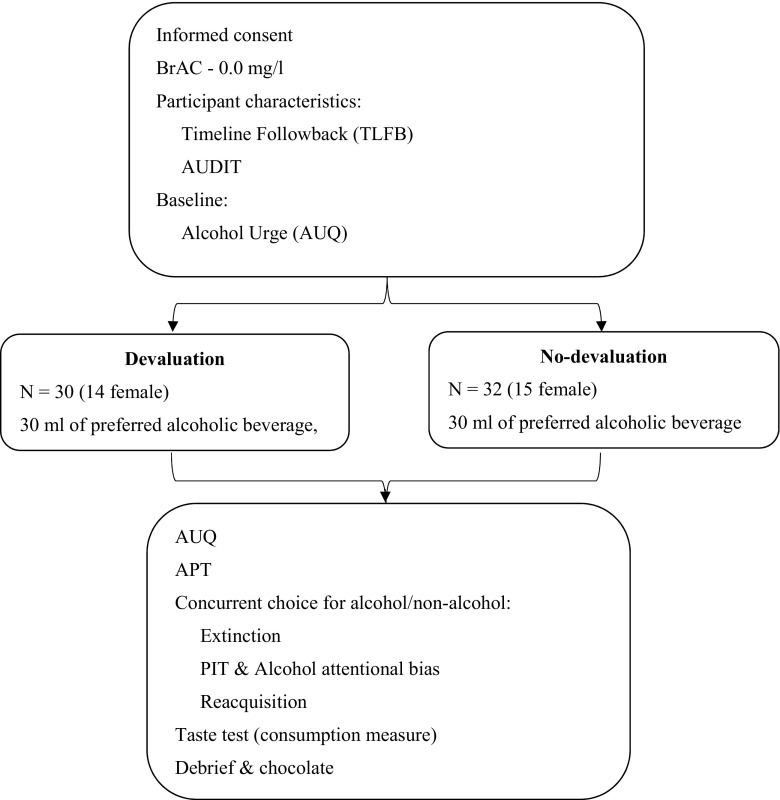
Schematic diagram of study procedure

All testing took place between 12 and 6 p.m. in the eye-tracking lab of the University of Liverpool. Participants provided informed consent and a breathalyser reading of 0.0 mg/l. Participants completed the AUDIT and TLFB, and baseline measures of alcohol craving and demand (AUQ and APT). They then completed the acquisition stage of the concurrent choice task, before consuming 30 ml of beer: half the participants received adulterated beer [devaluation group], whereas half received unadulterated beer [control group]. Immediately following the manipulation, participants completed the AUQ and APT for a second time before the extinction, PIT and reacquisition phases of the concurrent choice task. Participants completed the bogus taste test and were then provided with some chocolate to remove any bitter aftertaste before being debriefed (see Fig. 2).

## Results

### Participants

Independent *t* tests showed that the two groups were well-matched, with no pre-existing group differences (Table 1).

**Table 1:** Means (±SD) for participant age and drinking habits by group

	Group		
	Control	Devaluation	Statistics
Variable			
Age	22.75 (5.15)	21.93 (3.02)	*t* (60) = .75, *p* = .45
AUDIT	14.33 (5.69)	13.91 (4.73)	*t* (60) = −0.32, *p* = .75
Units per week	24.85 (17.76)	29.25 (14.95)	*t* (60) = 1.05, *p* = .30
Binges per week	1.54 (0.86)	1.25 (0.94)	*t* (60) = 1.30, *p* = .20

### Does reducing the value of alcohol affect motivation for and behaviour towards alcohol?

#### Alcohol Craving

Alcohol craving increased over time in the control group and this effect was supressed by devaluation (Table 2). A 2 (group: devaluation vs. control, between-subjects) by 2 (time: before vs. after devaluation, within-subjects) mixed design ANOVA yielded a significant main effect of time, *F*(1, 60) = 19.54, *p* < .001, that was subsumed by a significant interaction between the two, *F*(1,60) = 7.33, *p* = .001. There was no effect of group, *F* (1, 60) = 2.09, *p* = .153. Independent *t* tests showed that groups did not differ at baseline, *t*(60) = .42, *p* = .68, but did post-manipulation, *t*(60) = 2.35, *p* = .02, and that the time effect was reliable in the control group, *t*(31) = 5.12, *p* < .001, but not in the devaluation group, *t*(29) = 1.19, *p* = .24.

**Table 2:** Descriptive statistics (mean, ±SD) for alcohol urge and behavioural economic indices derived from the alcohol purchase task according to group and time

	Group	Time	
		Baseline	Post-manipulation
Variable			
Alcohol urge	Devaluation	16.57 (6.93)	18.47 (9.68)
	Control	15.91 (5.52)	23.81 (8.25)
Intensity	Devaluation	.47 (.35)	.05 (.87)
	Control	.52 (.29)	.55 (.31)
*O*_max_	Devaluation	.26 (.89)	− .16 (1.14)
	Control	.63 (.44)	.71 (.43)
*P*_max_	Devaluation	.02 (.80)	− .36 (1.03)
	Control	.29 (.36)	.33 (.32)
Breakpoint	Devaluation	.27 (.83)	− .15 (1.12)
	Control	.59 (.33)	.64 (.31)
Alpha (elasticity)	Devaluation	− 1.84 (.32)	− 1.74 (.30)
	Control	− 1.86 (.31)	− 1.91 (.30)

#### Alcohol purchase task

Alcohol devaluation decreased all indices of alcohol demand (Table 2). Data were log transformed to reduce positive skew. Four outliers were recoded to the next highest outlying value (Tabachnick and Fidel 2006). A 2 (group) by 2 (time) mixed ANOVA identified significant interactions for all APT factors, *F*s ≥ 5.67, *p*s ≤ .02. Scores on these factors decreased over time in the devaluation group *t*s ≥ 2.17, *p*s ≤ .04. Post-manipulation scores were lower in the devaluation, compared to the control, group *t*s ≥ 3.01, *p*s ≤ .004. The exception was that the post-manipulation score for elasticity was only marginally lower in the devaluation group, *t* (52) = 1.98, *p* = .05. See supplemental document, Tables 1 and 2, for inferential statistics for AUQ and APT.

#### Alcohol choice

##### Choice task

**Fig. 3:**
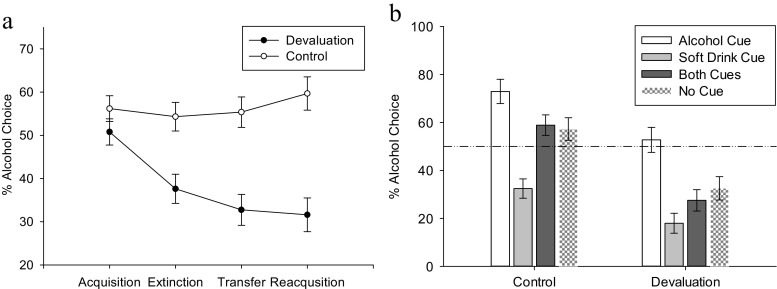
Left, the average proportion of alcohol responses made across the four phases of the choice task in the devaluation and control groups (M, ±SEM). Right, the average proportion of alcohol responses across the different trial types (based on the cues presented) of the transfer (PIT) phase of the choice task (M, ±SEM)

Alcohol devaluation decreased alcohol choice (Fig. 3). The effect of the manipulation on choice was assessed by comparing the proportion of alcohol, relative to soft drink, responses during each phase of the task. Degrees of freedom were corrected using Greenhouse-Geisser (GG) estimates (*ε* = .716) due to violation of sphericity, *χ*^2^(2) = 33.75*, p* < .001, (*W* = .557).

A two (group) by four (phase: acquisition, extinction, general PIT, reacquisition; within-subjects) mixed ANOVA found a main effect of group, *F*(1, 60) = 20.19, *p* < .001, *ŋ*_*p*_^*2*^ = .252; proportion of alcohol responses was lower in the devaluation group. There was also a main effect of phase *F*(2.15, 126.71) = 9,34, *p* < .001, *ŋ*_*p*_^*2*^ = .137, and a two way interaction between group and phase, *F*(2.15, 126.71) = 12.41, *p* < .001, *ŋ*_*p*_^*2*^ = .174. In the devaluation group, alcohol choice decreased between acquisition (i.e., baseline) and all post-manipulation phases, *ts* ≥ 4.20, *ps* ≤ .001. In the control group, alcohol choice did not change between acquisition and any post-manipulation phase, *ts* ≤ 1.02, *ps* = 1.000. During acquisition, alcohol choice did not differ between the two groups, *t*(59) = 1.27, *p* = .21, while during all post-manipulation phases, alcohol choice was lower in the devaluation group, *ts* ≥ 3.54, *ps* ≤ .001. The devaluation effect did not differ between the various test stages: extinction, PIT and reacquisition. See supplemental document, Tables 3 and 4, for means and post hoc inferential statistics.

##### Pavlovian to instrumental transfer (specific PIT)

Reducing alcohol value did not affect the ability of cues to prime choice of the corresponding response (Fig. 3). A two (group) by four (cue type: alcohol, soft drink, both, none; within-subjects) mixed ANOVA assessed proportion of alcohol responses across PIT trials. Degrees of freedom were corrected using GG estimates (*ε* = .760) due to violation of sphericity, *χ*^2^(2) = 26.969, *p* < .001, (*W* = .632). A main effect of group, *F*(1, 60) = 20.19, *p* < .001, *ŋ*_*p*_^*2*^ = .252: proportion of alcohol responses was lower in the devaluation group. A main effect of cue type, *F*(2.28, 136.81) = 41.90, *p* < .001, *ŋ*_*p*_^*2*^ = .411: alcohol responses were more likely on alcohol cue trials than other trial types, *t*s ≥ 5.32, *p*s < .001. Soft drink choice was more likely during soft drink trials than all other trials, *t*s ≥ 5.96, *p*s < .001. No cue and both cue trials did not differ, *t*(61) = .72, *p* > .99. Crucially, the interaction between cue type and group was *not* significant, *F*(2.280, 136.806) = 2.62, *p* = .101, *ŋ*_*p*_^*2*^ = .036, despite the reliable main effect. This indicates that devaluation did not reduce the ability of the alcohol cue to trigger alcohol choice above baseline. See supplemental document, Tables 5 and 6, for means and post hoc inferential statistics.

#### Attention

**Fig. 4:**
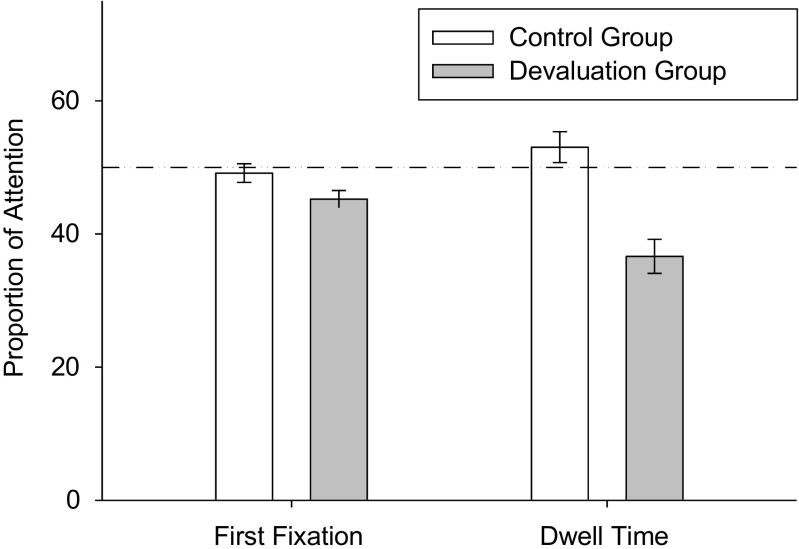
The proportion of attention towards alcohol cues when both an alcohol and soft drink cue were simultaneously presented in the transfer (PIT) phase of the choice task (M, ±SEM)

Alcohol devaluation supported an attentional preference for soft drink stimuli (Fig. 4). Data from the PIT both cue trials assessed alcohol attentional bias. Two participants were excluded due to lost data. Alcohol devaluation reduced the proportion of initial fixations to alcohol cues, *t*(58) = 2.03, *p* = .047, and percent dwell time on the alcohol cues, *t*(58) = 4.76, *p* < .001, compared to control. One sample *t* tests determined whether attention to cues differed from chance (50%). In the devaluation group, participants showed greater initial fixation, *t*(27) = 3.71, *p* = .001, and dwell time, *t*(27) = 5.22, *p* < .001, to the soft drink, highlighting preferential attention to the soft drink following the manipulation. In the control group, initial fixations, *t*(31) = .60, *p* = .55, and dwell time, *t*(31) = 1.31, *p* = .20, on alcohol cues did not differ from chance.

#### Ad libitum drink consumption

**Fig. 5:**
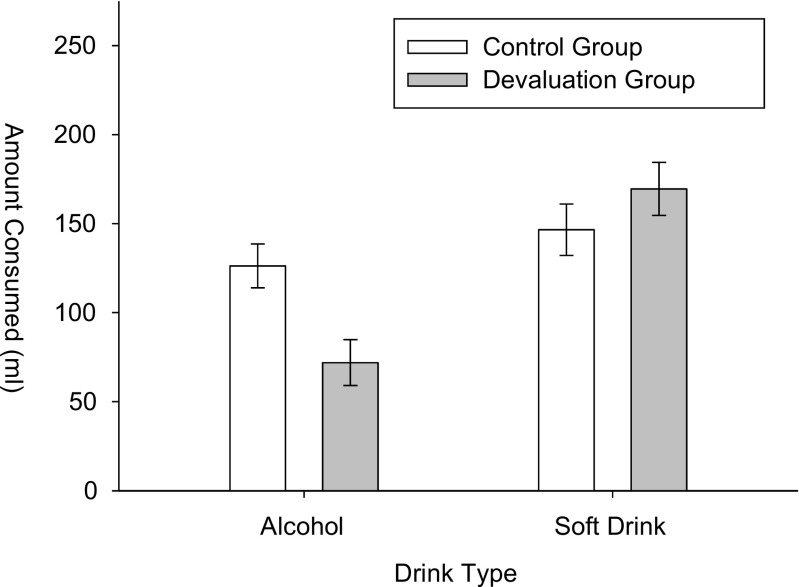
The amount of alcohol and soft drink consumed (ml) during the bogus taste test according to group (M, ±SEM)

Alcohol devaluation reduced alcohol, but not soft drink, consumption (Fig. 5). The mixed ANOVA (two [group] by two [drink type, within subject]) found a main effect of drink type *F*(1, 60) = 29.622, *p* < .001, *ŋp*^*2*^ = .331 and a significant interaction, *F*(1, 60) = 12.72, *p* = .001, *ŋ*_*p*_^*2*^ = .175. There was no main effect of group, *F*(1, 60) = .976, *p* = .327, *ŋp*^*2*^ = .016. Groups differed in alcohol, *t*(60) = 3.08, *p* = .003, but not soft drink, *t*(60) = 1.10, *p* = .275, consumption. The devalued group consumed less alcohol than soft drink, *t*(29) = 6.27, *p* < .001, whereas the control group consumed the two drinks comparably, *t*(31) = 1.35, *p* = .18.

With the exception of alcohol craving, the preceding analysis supports our first hypothesis. Devaluing alcohol decreases motivation for, and behaviour towards, alcohol, except cue-elicited alcohol responding.

### Do motivational processes mediate any effect of alcohol devaluation on alcohol behaviour?

Prior to conducting our planned mediation analyses, correlations were conducted to (a) identify the presence of collinear predictors and (b) determine the extent to which individual differences in self-report measures were related to indices of alcohol value and choice. See supplemental document, Table 7.

#### Mediation

Overall, mediation analysis showed that there were significant negative relationships between the devaluation manipulation and the experimental outcome variables (choice and consumption), with mediation effects of motivation indices differing according to outcome. The PROCESS macro for SPSS (Hayes 2013) was utilised to calculate: (1) the indirect relationship between the devaluation manipulation and alcohol choice via motivation indices and (2) the indirect relationship between the devaluation manipulation and alcohol consumption via motivation indices. AB data was taken from PIT trials which included both drink cues. Only intensity of demand, the strongest predictor of consumption was included, for parsimony and to prevent multicollinearity (See supplemental document, Tables 7 and 8 for correlations and regression.)

##### Goal-directed and cue-elicited responding

**Fig. 6:**
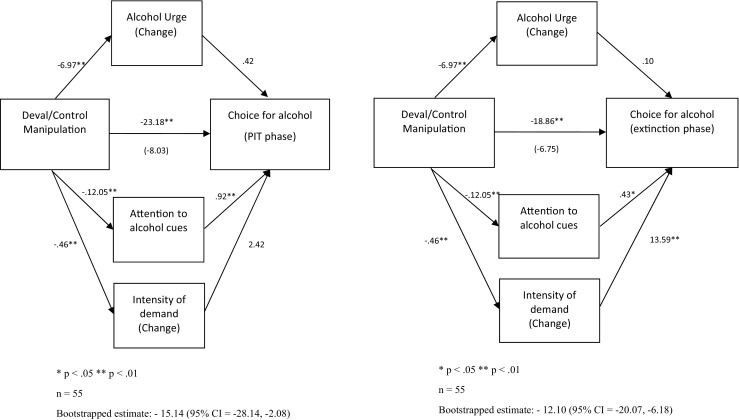
Left, path diagram highlighting the (full) mediation effect of attention on the relationship between the experimental manipulation and habitual choice for alcohol (PIT phase: alcohol cue-only trials). Right, path diagram highlighting the (full) mediation effect of attention and intensity of demand on the relationship between the experimental manipulation and goal-directed choice for alcohol (extinction phase)

Mediation analyses entered motivational indices (AB, changes in craving and intensity) as mediators between the devaluation manipulation and the proportion of alcohol choice during extinction (goal-directed behaviour), and during the alcohol cue trials of the PIT phase (cue-elicited behaviour) (Fig. 6). Intensity of demand and AB fully mediated goal-directed alcohol choice. AB fully mediated the effect of devaluation on cue-elicited alcohol choice.

##### Consumption

**Fig. 7:**
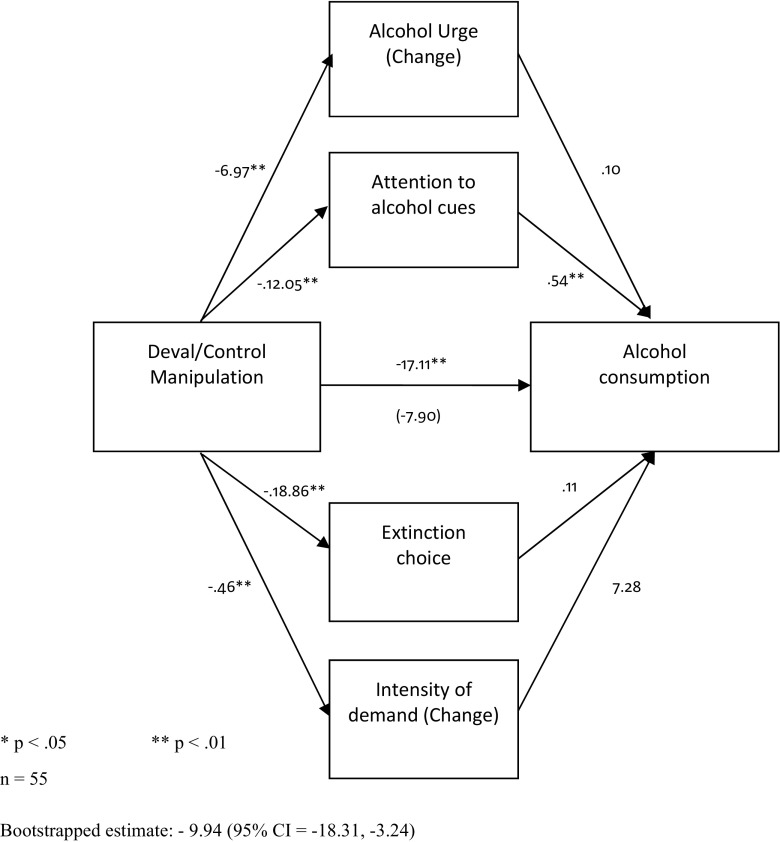
Path diagram highlighting the (full) mediation effect of attention on demand on the relationship between the experimental manipulation and alcohol consumption

Mediation analysis assessed whether the motivational components mediated the devaluation effect on alcohol consumption (Fig. 7). We included alcohol choice during extinction as the goal-directed measure of behaviour. Attentional bias fully mediated the relationship between the devaluation effect and consumption.

##### Replication

To clarify Rose et al.’s (2013) findings, we entered attention as the mediator between the devaluation manipulation and proportion of alcohol choice during the transfer phase. Attention towards alcohol cues partially mediated the effect of devaluation on choice. However, the *R*^2^ value highlighted that the indirect effect of attention accounted for 18% of the variance, lower than that found by Rose et al. (2013) (see supplemental document, Fig. 1).

## Discussion

The current study is the first to investigate the role alcohol value may have in goal-directed and cue-elicited alcohol seeking (choice), and self-administration (consumption) using a devaluation paradigm. Results show that changing the current value of alcohol decreased several components of alcohol motivation (AB and behavioural economic demand) and behaviour (goal-directed choice and consumption). The decrease in choice during the extinction phase, following devaluation, suggests that motivation for alcohol is commonly governed by goal-directed processes; that is, controlled by the current incentive value of alcohol. In addition, although there was an increase in craving in the control group, this effect was not seen after alcohol devaluation. The increase in craving in the control group was unexpected but, speculatively, may have been due to eliciting positive alcohol expectancies (from the unadulterated alcohol sip) without making alcohol immediately available (MacKillop and Lisman 2005).

Although we found that devaluation reduced overall alcohol choice during the PIT phase (which was conducted in extinction, indicating goal-directed choice), presentation of the alcohol stimulus primed alcohol choice above baseline, irrespective of devaluation group. This substantiates a variety of human and animal procedures which suggest that cue-elicited priming of drug-seeking is a dissociable form of behavioural control; separate from motivational indices such as AB, economic demand, and craving which were all sensitive to devaluation (e.g. Cartoni et al. 2013; Hitsman et al. 2013). Overall, our PIT effect provides human experimental support for the common anecdote that substance users persevere with drug use despite claiming they do not like the drug.

Although the current study did not set out to identify the mechanisms underlying outcome specific PIT, it is interesting to note that there are a number of theoretical claims concerning specific PIT. For example, that it occurs due to an expectancy that a particular response-outcome has a greater likelihood of being successful (Cartoni et al. 2013), that the cue activates a representation of the outcome’s sensory properties (but not current value) (Cohen-Hatton et al. 2013), that this effect involves greater input from model-free systems (Sebold et al. 2016), while others argue that model-based systems are involved (Huys et al. 2014). Future research needs to clarify the processes underlying specific PIT, and this research will undoubtedly involve further assessment of the role of current value. If, as we argue, value is a key driver in goal-directed drug behaviour, it would be important for future research to determine whether a negative relationship existed between PIT effects before and after devaluation, and incentive value-based decision-making and how this might map on to model-free and model-based systems.

Mediation analysis found that AB and behavioural economic demand for alcohol fully mediated the effect of devaluation on goal-directed alcohol choice. We would argue that the partial mediating role of economic demand in goal-directed choice supports the assertion that value is a causal factor in such behaviour (Field et al. 2016). In addition to partially mediating goal-directed choice, AB fully mediated the effect of alcohol devaluation on cue-elicited choice and consumption. Although some have argued for a causal role of AB on alcohol/drug behaviour (e.g., Franken 2003), a number of studies have either failed to support this claim or have not included adequate control conditions (for a review, see Field et al. 2016). The recent suggestion that AB is an output of the (causal) incentive value of the stimulus, and so AB will always be more predictive when it is measured at a point proximal to the behaviour (e.g. choice, consumption) and in the same environment (Field et al. 2016), is supported by the current study. In line with this, although well-practiced drug behaviours may favour habitual control (Tiffany 1990), the novel taste devaluation procedure used in the current study may have resulted in consumption that was entirely governed by the hedonic evaluation of the drinks, and thus was fully mediated by AB which reflects these incentive valuations.

Clearly, AB has a role to play in the expression of alcohol behaviour: it is an implicit measure which seems to be intrinsically associated with current incentive value (Anderson and Halpern 2017), perhaps subsuming more explicit, self-report measures of alcohol motivation (e.g. craving, which had no mediating role in the current study). Although we did not set out to determine whether implicit or explicit processes were stronger mediators of alcohol-related behaviour, this is an interesting question. The types of assessment used differed across outcomes: AB was measured through eye movement and can be categorised as an implicit measure, craving and economic demand was measured through self-report and would be categorised as more explicit measures (Wiers et al. 2007). Future research should identify, where applicable, whether implicit or explicit aspects of a cognitive process are more strongly associated with substance value, which we would argue is the core driver.

The finding that AB played a mediating role in both value- and stimulus-driven alcohol behaviour seems somewhat paradoxical. The finding that AB fully mediated the specific PIT effect in the current devaluation paradigm, indicates that the ability of a cue to trigger an outcome-specific response maybe positively associated with that cue’s ability to attract attention, regardless of current value. This supports the argument that during specific PIT, the cue activates sensory, but not motivational, properties of the drug (Corbit and Balleine 2016). Therefore, as well as being an indicator of value, it is possible that AB has multiple roles in different aspects of associative learning which future research should clarify.

There are several limitations to this study. We did not record participants’ beliefs regarding the study, e.g. did they believe they were responding to earn drinks to take home, did those in the devaluation condition know that the ‘take home drinks’ were unadulterated? These beliefs may have affected behaviour and future research should control for these possibilities. Additionally, we cannot apply our findings to dependent substance users. Future work should evaluate a similar paradigm in a clinically dependent population. Although it is worth noting that our sample were heavy drinkers who consumed ~ 27 units a week, binged weekly, and scored an average of 14 on the AUDIT (indicating hazardous drinking), and so the study provides data on a potentially critical transitional stage of hazardous drinking. In addition, there was no effect of individual differences (e.g. AUDIT score) on the devaluation effect or specific PIT effects (see supplemental document, Table 7). This is supported by existing literature (for a review, see Hogarth et al. 2013a) and suggests that these associative learning mechanisms are important in understanding alcohol-related behaviour regardless of severity.

A secondary aim of this paper was to replicate our earlier finding that AB partially mediated the effect of devaluation on alcohol choice (Rose et al. 2013). Specifically, we found that AB to alcohol accounted for ~ 18% of the variance in the effect of alcohol devaluation on choice. This is a lower estimate (18 vs. 30%) than our previous work but is more in line with previous findings of ~ 10% (Armel et al. 2008; Krajbich et al. 2010). We suggest that the current finding is a more valid estimate given that Rose et al. (2013) required choice responses based on cue location, which likely inflated the association between attention and choice.

In terms of treatment implications, if future research supports our finding that drug behaviours are the culmination of two key processes (incentive value, stimulus influence) then combined treatments which target these two aspects may be effective. Although several pharmacotherapies may work through reducing alcohol’s value (e.g. naltrexone, disulfiram; e.g. Bujarski et al. 2012), the difficulty comes in finding practical psychosocial devaluation techniques. Although taste aversion and specific satiety are valid lab-based methods, they cannot practically be applied as interventions/treatments. Reducing value by highlighting the negative aspects of alcohol is a possibility but research shows that the effectiveness of such initiatives often work through ‘intervening variables’, e.g. campaigns stimulate conversations about alcohol’s negative effects which then reduces intention to drink (Hendriks et al. 2012). In addition, the control that cues exert over behaviour could be tackled within learning-based treatments. Rather than attempting to extinguish cue-based associations, which have previously resulted in disappointing treatment results (Conklin and Tiffany 2002), we would suggest using cognitive behavioural techniques. These can help the individual understand the autonomous effects drug stimuli have on drug seeking (Monti et al. 2002) and teach skills to resist the effects. Alternatively, pharmacological enhancement of extinction-based procedures and/or disruption of reconsolidation of Pavlovian associations may also have promise (MacKillop et al. 2015; Milton et al. 2012). Research has found that AB is not a useful predictor of treatment outcome, and that attentional retraining is not an effective treatment (e.g. Cristea et al. 2016). However, if AB is a measurable index of current value, it may be possible to train patients to identify when they are experiencing AB and to understand this as a warning sign of potential relapse, and to act accordingly (Field et al. 2016). Additionally, as PIT tends to trigger approach responses to appetitive stimuli, other forms of cognitive bias modification (CBM) techniques may be useful. For example, Wiers et al. (2011) found better treatment outcomes 1 year after patients received CBM aimed at reducing alcohol approach bias. Given the involvement of multiple processes on alcohol-related behaviour, such combined treatment regimens are worthy of future research.

In conclusion, the current findings suggest that alcohol motivation and behaviour are sensitive to devaluation and are therefore predominantly governed by value-based decision-making. However, a separate form of stimulus control also drives alcohol choice irrespective of current value. Although the mechanisms that drive specific PIT remain to be confirmed it is likely to involve the cue increasing the expected probability of the response being rewarded. Indices of motivation (e.g. AB, craving, economic demand) can all be understood as measures of the causal factor of value. However, paradoxically, AB may mediate both value- and stimulus-based forms of behaviour control. Importantly, these results provide new perspectives on the basic mechanisms underlying alcohol-related motivation and behaviour and suggest that combined treatments which target these mechanisms may be effective in reducing hazardous drinking.

## Electronic supplementary material

ESM 1 (DOCX 62 kb)
