# Supplementary material for: Alcohol devaluation has dissociable effects on distinct components of alcohol behaviour
Source: Psychopharmacology (Berl). 2018 Feb 26;235(4):1233–44. doi: 10.1007/s00213-018-4839-2 (PMC5869941; doi:10.1007/s00213-018-4839-2)
Supplement: Supplementary file 1 — (DOCX 62 kb) [file 213_2018_4839_MOESM1_ESM.docx]

**Supplemental Document**

Alcohol Urge & Alcohol Purchase Task (see Table 1 & 2)

|  | **Test** |  | ***df*** |  | ***F*** |  | **η_p_^2^** |  | **p** |
| --- | --- | --- | --- | --- | --- | --- | --- | --- | --- |
| **Variable** |  |  |  |  |  |  |  |  |  |
| Alcohol Urge | (A) Time |  | 1 |  | 19.54 |  | .25 |  | **<.001** |
|  | (B) Group |  | 1 |  | 2.09 |  | .03 |  | .153 |
|  | A x B (Interaction) |  | 1 |  | 7.33 |  | .11 |  | **.009** |
|  | Error (Within Groups) |  | 60 |  |  |  |  |  |  |
| Intensity | (A) Time |  | 1 |  | 10.18 |  | .16 |  | **.002** |
|  | (B) Group |  | 1 |  | 5.65 |  | .09 |  | **.021** |
|  | A x B (Interaction) |  | 1 |  | 13.34 |  | .20 |  | **.001** |
|  | Error (Within Groups) |  | 55 |  |  |  |  |  |  |
| Omax | (A) Time |  | 1 |  | 4.84 |  | .08 |  | **.032** |
|  | (B) Group |  | 1 |  | 10.79 |  | .16 |  | **.002** |
|  | A x B (Interaction) |  | 1 |  | 10.30 |  | .16 |  | **.002** |
|  | Error (Within Groups) |  | 55 |  |  |  |  |  |  |
| Pmax | (A) Time |  | 1 |  | 4.15 |  | .07 |  | **.046** |
|  | (B) Group |  | 1 |  | 9.56 |  | .15 |  | **.003** |
|  | A x B (Interaction) |  | 1 |  | 5.97 |  | .10 |  | **.018** |
|  | Error (Within Groups) |  | 55 |  |  |  |  |  |  |
| Breakpoint | (A) Time |  | 1 |  | 4.91 |  | .08 |  | **.031** |
|  | (B) Group |  | 1 |  | 11.04 |  | .17 |  | **.002** |
|  | A x B (Interaction) |  | 1 |  | 7.76 |  | .12 |  | **.007** |
|  | Error (Within Groups) |  | 55 |  |  |  |  |  |  |
| Alpha (elasticity) | (A) Time |  | 1 |  | .84 |  | .02 |  | .362 |
|  | (B) Group |  | 1 |  | 1.32 |  | .03 |  | .255 |
|  | A x B (Interaction) |  | 1 |  | 5.67 |  | .01 |  | **.021** |
|  | Error (Within Groups) |  | 51 |  |  |  |  |  |  |

Table 1: Inferential Statistics (Main effects, Interactions) for alcohol urge and behavioural economic indices derived from the alcohol purchase task according to time and group. All interactions were significant.

Table 2: Inferential Statistics (Post Hoc) for alcohol urge and behavioural economic indices according to group and time.

Shows that 1) there were no group differences in baseline on any urge or demand measures, 2) alcohol urge increased after the sip prime in the control but not devaluation group, 3) measures of economic demand for alcohol decreased following alcohol devaluation but did not change in the control group. This suggests that reduction of alcohol value blocked any increase in urge for alcohol and decreased economic demand for alcohol.

|  | **Group** | **Test** |  | ***df*** |  | ***t*** |  | **p** |
| --- | --- | --- | --- | --- | --- | --- | --- | --- |
| **Variable** |  |  |  |  |  |  |  |  |
| Alcohol Urge | Devaluation | Baseline v Post |  | 29 |  | 1.192 |  | .238 |
|  | Control | Baseline v Post |  | 31 |  | 5.123 |  | **<.001** |
|  | Baseline | Devaluation v Control |  | 60 |  | .416 |  | .679 |
|  | Post | Devaluation v Control |  | 60 |  | 2.346 |  | .**022** |
|  |  |  |  |  |  |  |  |  |
| Intensity | Devaluation | Baseline v Post |  | 25 |  | 4.642 |  | **<.001** |
|  | Control | Baseline v Post |  | 30 |  | .337 |  | .734 |
|  | Baseline | Devaluation v Control |  | 55 |  | .654 |  | .516 |
|  | Post | Devaluation v Control |  | 56 |  | 3.001 |  | **.004** |
|  |  |  |  |  |  |  |  |  |
| Omax | Devaluation | Baseline v Post |  | 25 |  | 3.672 |  | **.001** |
|  | Control | Baseline v Post |  | 30 |  | .746 |  | .459 |
|  | Baseline | Devaluation v Control |  | 55 |  | 1.880 |  | .065 |
|  | Post | Devaluation v Control |  | 56 |  | 3.941 |  | **<.001** |
|  |  |  |  |  |  |  |  |  |
| Pmax | Devaluation | Baseline v Post |  | 25 |  | 3.031 |  | **.004** |
|  | Control | Baseline v Post |  | 30 |  | .300 |  | .765 |
|  | Baseline | Devaluation v Control |  | 55 |  | 1.706 |  | .094 |
|  | Post | Devaluation v Control |  | 56 |  | 3.534 |  | **.001** |
|  |  |  |  |  |  |  |  |  |
| Breakpoint | Devaluation | Baseline v Post |  | 25 |  | 3.396 |  | **.001** |
|  | Control | Baseline v Post |  | 30 |  | .424 |  | .673 |
|  | Baseline | Devaluation v Control |  | 55 |  | 1.96 |  | .055 |
|  | Post | Devaluation v Control |  | 56 |  | 3.775 |  | **<.001** |
|  |  |  |  |  |  |  |  |  |
| Alpha (elasticity) | Devaluation | Baseline v Post |  | 21 |  | 2.154 |  | **.036** |
|  | Control | Baseline v Post |  | 30 |  | 1.133 |  | .262 |
|  | Baseline | Devaluation v Control |  | 55 |  | .184 |  | .854 |
|  | Post | Devaluation v Control |  | 52 |  | 1.991 |  | .052 |

Alcohol Choice (see Table 3 & 4)

Table 3 shows descriptives for alcohol choice during the four phases of the PIT choice task. Table 3 shows: 1) no difference in alcohol choice at baseline between the two groups, 2) no difference in proportion of alcohol choice across any of the different choice phases in the control group, 3) that compared with acquisition (i.e., baseline), choice was lower in all post-manipulation choice phases in the devaluation group. These data indicate that the devaluation manipulation directly decreased choice for alcohol, and that this effect was not significantly moderated by the presentation of cues or feedback.

|  | **Group** | **M** |  | ***SD*** |  | ***SE*** |
| --- | --- | --- | --- | --- | --- | --- |
| **Phase** |  |  |  |  |  |  |
| Acquisition | Control | 56.18 |  | 18.82 |  | 3.38 |
|  | Devaluation | 50.78 |  | 13.89 |  | 2.54 |
|  |  |  |  |  |  |  |
| Extinction | Control | 54.08 |  | 14.44 |  | 2.55 |
|  | Devaluation | 37.62 |  | 21.72 |  | 3.97 |
|  |  |  |  |  |  |  |
| Transfer | Control | 55.13 |  | 16.36 |  | 2.89 |
|  | Devaluation | 32.75 |  | 22.23 |  | 4.06 |
|  |  |  |  |  |  |  |
| Reacquisition | Control | 59.31 |  | 18.27 |  | 3.23 |
|  | Devaluation | 31.60 |  | 24.10 |  | 4.40 |

Table 3: Descriptive statistics highlighting the proportion of responses for alcohol (relative to soft drinks), according to experimental condition, during the different phases of the choice task (M, ±SD, SE).

| **Group** | **Test** |  | ***df*** |  | ***t*** |  | **p** |
| --- | --- | --- | --- | --- | --- | --- | --- |
| Control | Acquisition v Extinction |  | 31 |  | 0.606 |  | 1.000 |
|  | Acquisition v Transfer |  | 31 |  | 0.250 |  | 1.000 |
|  | Acquisition v Reacquisition |  | 31 |  | 1.022 |  | 1.000 |
|  | Extinction v Transfer |  | 32 |  | 0.565 |  | 1.000 |
|  | Extinction v Reacquisition |  | 32 |  | 2.341 |  | .136 |
|  | Transfer v Reacquisition |  | 32 |  | 2.115 |  | .232 |
|  |  |  |  |  |  |  |  |
| Devaluation | Acquisition v Extinction |  | 30 |  | 4.196 |  | **.001** |
|  | Acquisition v Transfer |  | 30 |  | 5.400 |  | **.000** |
|  | Acquisition v Reacquisition |  | 30 |  | 5.516 |  | **.000** |
|  | Extinction v Transfer |  | 30 |  | 2.583 |  | .074 |
|  | Extinction v Reacquisition |  | 30 |  | 2.584 |  | .073 |
|  | Transfer v Reacquisition |  | 30 |  | 0.554 |  | 1.000 |
|  |  |  |  |  |  |  |  |
| Acquisition | Control v Devaluation |  | 59 |  | 1.273 |  | .208 |
| Extinction | Control v Devaluation |  | 60 |  | 3.532 |  | **.001** |
| Transfer | Control v Devaluation |  | 60 |  | 4.515 |  | **.000** |
| Reacquisition | Control v Devaluation |  | 60 |  | 5.120 |  | **.000** |

Table 4: Inferential Statistics (Post Hoc) for group x phase interaction on proportion of alcohol choice.

PIT Choice

Table 5 shows descriptives for alcohol choice during just the transfer phase of the PIT choice task, separated by cue trial type.

Table 6 shows that 1) alcohol choice was more likely during alcohol cue trials compared with all other trial types, 2) soft drink choice was more likely during soft drink cue trails compared with Both/No cue trials, 3) there was not difference in alcohol choice between both cue and no cue trials.

These data indicate that, irrespective of devaluation, presentation of a reward-related cue increases the likelihood of choosing the corresponding reward

|  | **Group** | **M** |  | ***SD*** |  | ***SE*** |
| --- | --- | --- | --- | --- | --- | --- |
| **Phase** |  |  |  |  |  |  |
| Alcohol Only | Control | 72.97 |  | 22.96 |  | 4.06 |
|  | Devaluation | 52.78 |  | 33.36 |  | 6.09 |
|  |  |  |  |  |  |  |
| Soft drink Only | Control | 32.45 |  | 22.47 |  | 3.97 |
|  | Devaluation | 18.00 |  | 23.20 |  | 4.24 |
|  |  |  |  |  |  |  |
| Both | Control | 58.91 |  | 23.43 |  | 4.14 |
|  | Devaluation | 27.56 |  | 25.25 |  | 4.61 |
|  |  |  |  |  |  |  |
| No cues | Control | 57.29 |  | 22.69 |  | 4.01 |
|  | Devaluation | 32.56 |  | 30.14 |  | 5.50 |

Table 5: Descriptive statistics highlighting the proportion of responses for alcohol and soft drink cues during the presentation of different cue combinations in the PIT stage of the choice task (M, ±SD, SE).

| **Test** |  | ***df*** |  | ***t*** |  | **p** |
| --- | --- | --- | --- | --- | --- | --- |
| Alcohol cue v Soft drink cue |  | 61 |  | 8.415 |  | **<.001** |
| Alcohol cue v Both cues |  | 61 |  | 5.905 |  | **<.001** |
| Alcohol cue v No cues |  | 61 |  | 5.317 |  | **<.001** |
| Soft drink cue v Both cues |  | 61 |  | 5.957 |  | **<.001** |
| Soft drink cue v No cues |  | 61 |  | 6.055 |  | **<.001** |
| Both cues v No cues |  | 61 |  | 0.716 |  | 1.000 |

Table 6: Inferential Statistics (Post Hoc) for main effect of cue type on proportion of alcohol choice

**Mediation Analysis**

Table 7 shows correlations between all motivational components and alcohol-related behaviour. Table 8 shows regression results, and the selection of Intensity as the index for alcohol economic demand index.

|  | **Group** | **Cons.** | **Urge** | **AUDIT** | **TLFB (Wk)** | **Acq.** | **Ext.** | **Reacq.** | **Trans. (Ave)** | **Trans. (Alc)** | **Trans. (Soft)** | **Deval.** | **Att.** | **BP** | **Intens.** | **Alpha** | **Omax** | **Pmax** |
| --- | --- | --- | --- | --- | --- | --- | --- | --- | --- | --- | --- | --- | --- | --- | --- | --- | --- | --- |
| **Group** | 1 |  |  |  |  |  |  |  |  |  |  |  |  |  |  |  |  |  |
| **Consumption** | -.502^**^ | 1 |  |  |  |  |  |  |  |  |  |  |  |  |  |  |  |  |
| **Alcohol Urge** | -.330^**^ | .227 | 1 |  |  |  |  |  |  |  |  |  |  |  |  |  |  |  |
| **AUDIT** | .041 | -.198 | .040 | 1 |  |  |  |  |  |  |  |  |  |  |  |  |  |  |
| **TLFB (Week)** | -.135 | -.033 | .245 | .617^**^ | 1 |  |  |  |  |  |  |  |  |  |  |  |  |  |
| **Acquisition** | -.163 | .273^*^ | .179 | .323^*^ | .475^**^ | 1 |  |  |  |  |  |  |  |  |  |  |  |  |
| **Extinction** | -.415^**^ | .497^**^ | .411^**^ | .018 | .105 | .538^**^ | 1 |  |  |  |  |  |  |  |  |  |  |  |
| **Reacquisition** | -.552^**^ | .619^**^ | .345^**^ | .105 | .251^*^ | .519^**^ | .840^**^ | 1 |  |  |  |  |  |  |  |  |  |  |
| **Transfer (Average)** | -.505^**^ | .527^**^ | .394^**^ | .065 | .175 | .506^**^ | .880^**^ | .891^**^ | 1 |  |  |  |  |  |  |  |  |  |
| **Transfer (Alcohol)** | .087 | .024 | -.005 | -.136 | -.110 | -.077 | -.093 | -.105 | -.092 | 1 |  |  |  |  |  |  |  |  |
| **Transfer (Soft)** | -.200 | .377^**^ | .230 | -.020 | .100 | .306^*^ | .310^*^ | .413^**^ | .361^**^ | -.105 | 1 |  |  |  |  |  |  |  |
| **Overall Devaluation** | -.456^**^ | .425^**^ | .300^*^ | -.207 | -.200 | -.257^*^ | .610^**^ | .646^**^ | .665^**^ | -.052 | .170 | 1 |  |  |  |  |  |  |
| **Attention** | -.406^**^ | .575^**^ | .215 | .132 | .105 | .314^*^ | .505^**^ | .659^**^ | .592^**^ | -.182 | .405^**^ | .468^**^ | 1 |  |  |  |  |  |
| **BP** | -.352^**^ | .477^**^ | .381^**^ | -.023 | .087 | .164 | .362^**^ | .442^**^ | .352^**^ | .264^*^ | .161 | .314^*^ | .478^**^ | 1 |  |  |  |  |
| **Intensity** | -.442^**^ | .482^**^ | .389^**^ | -.009 | .057 | .314^*^ | .605^**^ | .541^**^ | .499^**^ | -.132 | .240 | .364^**^ | .469^**^ | .365^**^ | 1 |  |  |  |
| **Alpha** | .316^*^ | -.325^*^ | -.333^*^ | .090 | -.076 | -.004 | -.211 | -.341^*^ | -.333^*^ | .012 | -.172 | -.327^*^ | -.216 | -.382^**^ | -.662^**^ | 1 |  |  |
| **Omax** | -.397^**^ | .508^**^ | .381^**^ | -.023 | .103 | .209 | .408^**^ | .489^**^ | .392^**^ | .254 | .210 | .326^*^ | .493^**^ | .981^**^ | .384^**^ | -.475^**^ | 1 |  |
| **Pmax** | -.313^*^ | .389^**^ | .398^**^ | -.024 | .102 | .159 | .328^*^ | .397^**^ | .314^*^ | .301^*^ | .141 | .272^*^ | .432^**^ | .962^**^ | .329^*^ | -.344^*^ | .948^**^ | 1 |

* p < .05 ** p < .01

Table 7. Correlations between devaluation manipulation, choice across PIT stages (Transfer alcohol: percent alcohol choice in the alcohol stimulus trials - percent alcohol choice in the blank stimulus trials; Transfer soft:  percent soft choice in the blank stimulus trials - percent soft choice in the soft stimulus trials; Overall devaluation effect: average alcohol response across all tests – Baseline alcohol response (acquisition) and motivational indices (Urge [difference scores], attention and alcohol demand [difference scores])

|  |  |  | Collinearity | |
| --- | --- | --- | --- | --- |
|  | B | 95% CI | Tolerance | VIF |
| Breakpoint (BP) | 22.46 | [-15.55 – 60.47] | 0.03 | 30.93 |
| Intensity | 47.03** | [15.73 – 78.32] | 0.43 | 2.35 |
| Alpha | 9.71 | [-13.03 – 32.44] | 0.52 | 1.92 |
| Omax | 3.30 | [-34.21 - 40.80] | 0.03 | 30.95 |
| Pmax | -18.88 | [-39.72 – 1.97] | 0.10 | 9.88 |

Table 8. Regression depicting the relationship between alcohol demand indices and consumption, with measures of multicollinearity

Figure 1: Path diagram highlighting that attention partially mediates the relationship between the experimental manipulation and choice for alcohol in the transfer phase.

Deval/Control

Manipulation

Attention to alcohol cues

Choice for alcohol (transfer phase)

(-21.23**)

-31.41**

.91**

-11.22**

* p < .05 ** p < .01

n = 60

R^2^ = .18

Bootstrapped estimate: - 10.17 (95% CI = -19.63, -4.12)
